# Association of Delirium with Increased Risk of Mild Cognitive Impairment and Alzheimer’s Disease and Related Dementias

**DOI:** 10.1164/rccm.202412-2449RL

**Published:** 2025-04-10

**Authors:** Yubo Feng, Chao Yan, Yuankai Huo, Bingshan Li, Zhongliang Zu, Michael C. Smith, Mayur B. Patel, You Chen

**Affiliations:** ^1^ Department of Computer Science and; ^2^ Department of Molecular Physiology and Biophysics, Vanderbilt Genetics Institute, Vanderbilt University, Nashville, Tennessee;; ^3^ Department of Biomedical Informatics,; ^4^ Vanderbilt University Institute of Imaging Science,; ^5^ Division of Acute Care Surgery, Department of Surgery, Department of Neurosurgery, and Department of Hearing and Speech Sciences, Section of Surgical Sciences, and; ^6^ Critical Illness, Brain Dysfunction, and Survivorship Center, Center for Health Services Research, Vanderbilt University Medical Center, Nashville, Tennessee

*To the Editor*:

Delirium is an acute disturbance in consciousness and cognition often seen in hospitalized patients (1), linked to higher morbidity, prolonged stays, and increased costs (2, 3). Alzheimer’s disease and related dementias (ADRD) involve progressive cognitive and behavioral decline, impairing daily functioning (4). Although ICU delirium has been tied to later cognitive decline (5), the association of delirium throughout hospitalization (both ICU and non-ICU) with posthospital ADRD risk is unclear. The role of age and whether delirium accelerates ADRD onset also remain understudied. Using three decades of electronic health record (EHR) data from Vanderbilt University Medical Center, we investigated the age-stratified relationship between hospital-acquired delirium and subsequent mild cognitive impairment (MCI) and ADRD and whether delirium advances ADRD onset.

## Methods

After excluding patients with MCI/ADRD, stroke, Parkinson’s disease, or brain cancer before their first delirium episode, we identified 14,367 patients. We used a logistic regression model to estimate propensity scores for having a first delirium episode versus no delirium, including sex, race/ethnicity, prebaseline Charlson Comorbidity Index), and baseline measures shown in Table 1. We matched delirium cases to two control subjects by first applying exact matches on sex, race/ethnicity, type 2 diabetes, cardiovascular disease, sepsis, ICU admission, ventilator use, and missing respiratory rate. We then matched baseline visit year and age within 1 year, baseline visit length within 7 days, and prebaseline Charlson Comorbidity Index within 5 points. Finally, we selected two control subjects whose propensity scores were within 0.1 of each delirium case. The first two matching steps reduced the delirium group to 11,445, and the final step yielded 10,716 patients. Restricting index date to before January 1, 2020, left 7,670 patients, and excluding those who died or were discharged to hospice at baseline resulted in 6,557 delirium cases and 13,114 matched control subjects, all free of MCI/ADRD, stroke, Parkinson’s disease, or brain cancer at baseline. Our retrospective cohort, with index dates from 1990 to 2020, was followed through 2024, ensuring at least three years of observation for patients with delirium events in 2020. Both groups had baseline inpatient encounters.

**Table 1.** Summary Statistics of Delirium and Matched Control Groups

Characteristic	Delirium	Control	
	(*n* = 6,557)	(*n* = 13,114)	*P* Value
Sex, *n* (%)*			>0.999^†^
Female	2,848 (43.43)	5,696 (43.43)	>0.999^‡^
Male	3,709 (56.57)	7,418 (56.57)	>0.999^‡^
Race and ethnicity, *n* (%)*			>0.999^†^
White	5,857 (89.32)	11,714 (89.32)	>0.999^‡^
Black	572 (8.72)	1,144 (8.72)	>0.999^‡^
Other^§^	128 (1.95)	256 (1.95)	>0.999^‡^
T2DM at baseline, *n* (%)*			>0.999^†^
Yes	944 (14.4)	1,888 (14.4)	>0.999^‡^
No	5,613 (85.6)	11,226 (85.6)	>0.999^‡^
CVD at baseline, *n* (%)*			>0.999^†^
Yes	579 (8.83)	1,158 (8.83)	>0.999^‡^
No	5,978 (91.17)	11,956 (91.17)	>0.999^‡^
Within baseline visit, *n* (%)*			
Sepsis	671 (10.23)	1,342 (10.23)	>0.999^‡^
ICU	1,690 (25.77)	3,380 (25.77)	>0.999^‡^
Ventilator	1,273 (19.41)	2,546 (19.41)	>0.999^‡^
Missing respiratory rate	5,194 (79.21)	10,388 (79.21)	>0.999^‡^
Outcomes, *n* (%)			
ADRD	315 (4.8)	236 (1.8)	**<0.001** ^‡^
MCI	364 (5.55)	329 (2.51)	**<0.001** ^‡^
Death	1,033 (15.75)	1,601 (12.21)	**<0.001** ^‡^
Age category, *n* (%)			0.996^‡^
<30 yr	390 (5.95)	790 (6.02)	0.832^‡^
30–39 yr	523 (7.98)	1,019 (7.77)	0.613^‡^
40–49 yr	960 (14.64)	1,935 (14.76)	0.831^‡^
50–59 yr	1,329 (20.27)	2,668 (20.34)	0.900^‡^
60–69 yr	1,444 (22.02)	2,849 (21.72)	0.634^‡^
70–79 yr	1,140 (17.39)	2,291 (17.47)	0.884^‡^
≥80 yr	771 (11.76)	1,562 (11.91)	0.755^‡^
Prebaseline comorbidity, *n* (%)			
Hypertension	1,763 (26.89)	3,208 (24.46)	**<0.001** ^‡^
Dyslipidemia	202 (3.08)	369 (2.81)	0.293^‡^
Ischemic heart disease	756 (11.53)	1,530 (11.67)	0.777^‡^
Depression	588 (8.97)	767 (5.85)	**<0.001** ^‡^
Age at baseline, yr, mean ± SD*	59.01 (17.00)	59.04 (17.00)	0.888^‖^
ADI at baseline, mean ± SD*	55.38 (20.81)	57.58 (19.94)	**<0.001** ^‖^
CCI prebaseline, mean ± SD*	1.47 (2.41)	1.44 (2.21)	**0.003** ^‖^
Respiratory rate at baseline, breaths/min, mean ± SD*	14.67 (8.54)	14.19 (8.07)	**<0.001** ^‖^
Baseline visit length, d, mean ± SD*	9.37 (6.91)	8.67 (7.14)	**<0.001** ^‖^
Duration of delirium (postbaseline), d, mean ± SD	9.34 (6.88)	—	—
Follow-up years, mean ± SD			
Until ADRD	2.73 (3.91)	4.17 (4.54)	**<0.001** ^‖^
Until MCI	2.34 (3.90)	3.84 (4.60)	**<0.001** ^‖^
Until death	3.49 (4.16)	4.05 (4.64)	**0.018** ^‖^
Until censoring	13.1 (5.38)	12.92 (5.48)	0.072^‖^
Propensity score–matched subgroups comprising patients with discharge disposition information	*n* = 1,148	*n* = 2,296	
Discharge disposition, *n* (%)			
Home	629 (54.79)	1,690 (73.61)	**<0.001** ^‡^
Long-term care	334 (29.09)	390 (16.99)	**<0.001** ^‡^
Short-term care	179 (15.59)	211 (9.19)	**<0.001** ^‡^
Other	7 (0.61)	13 (0.57)	0.874^‡^

*Definition of abbreviations*: ADI = area deprivation index; ADRD = Alzheimer’s disease and related dementias; CCI = Charlson Comorbidity Index; CVD = cardiovascular disease; MCI = mild cognitive impairment; PSM = propensity score matching; T2DM = type 2 diabetes mellitus.

The *P* value column shows the results of statistical tests comparing each characteristic between the delirium and control groups.

* Factor we used in PSM.

^†^ Chi-Square Test.

^‡^ Proportion Test.

^§^ American Indian or Alaska Native, Asian, Hispanic or Latino, Native Hawaiian or Other Pacific Islander.

^‖^ Mann-Whitney U Test.

Delirium cases were identified from EHR data using the Observational Health Data Sciences and Informatics (OHDSI) phenotype library (6) (concept identifier 3739953). To focus solely on non–dementia-related delirium, we excluded records corresponding to concept identifiers 376946, 376094, and 381832. Because this study covers a diverse patient population over three decades, it required a standardized approach. OHDSI, an international collaborative consortium, supports mapping of delirium-related EHR data to standardized concept identifiers through algorithms guided by expert review and empirical validation, ensuring consistent and reliable large-scale analyses. In addition, this study uses widely recognized phenotyping algorithms to identify MCI and ADRD (7). A case of ADRD was confirmed if a patient’s EHR contained at least two postbaseline visits, with each visit recording any of the following International Classification of Diseases, Ninth and Tenth Revision, codes: Alzheimer’s disease (331.0, G30.0, G30.1, G30.8, and G30.9), vascular dementia (290.40, 290.41, F01.50, and F01.51), Lewy body dementia (331.82 and G31.83), frontotemporal dementia (331.19 and G31.09), and primary progressive aphasia (331.11 and G31.01). Similarly, an MCI diagnosis was confirmed using the code set 331.83, 294.9, G31.84, and F09.

We used Kaplan-Meier survival analysis and the log-rank test to compare probabilities of freedom from ADRD/MCI over time between the delirium and control groups. We used Fine-Gray (FG) subdistribution hazard models, adjusted for sex, race/ethnicity, baseline variables, and prebaseline comorbidities (Table 1), and accounted for death as a competing risk to compare overall MCI/ADRD incidence between delirium and control groups across all age categories. We also performed subgroup analyses by age category. Using the same covariates as in the FG models, we then calculated the restricted mean survival time (RMST) to assess differences in time to MCI/ADRD onset.

Because delirium duration may be associated with MCI/ADRD risk, we conducted an FG analysis within the delirium group, using the number of delirium days as the main variable and retaining the same covariates as the previous FG models. Given the importance of discharge disposition, and its high rate of missing data, we created delirium and control subgroups using the same propensity score matching approach for patients with available discharge disposition (Table 1), then repeated the analysis with discharge disposition included as a covariate.

## Results

**Figure 1.**
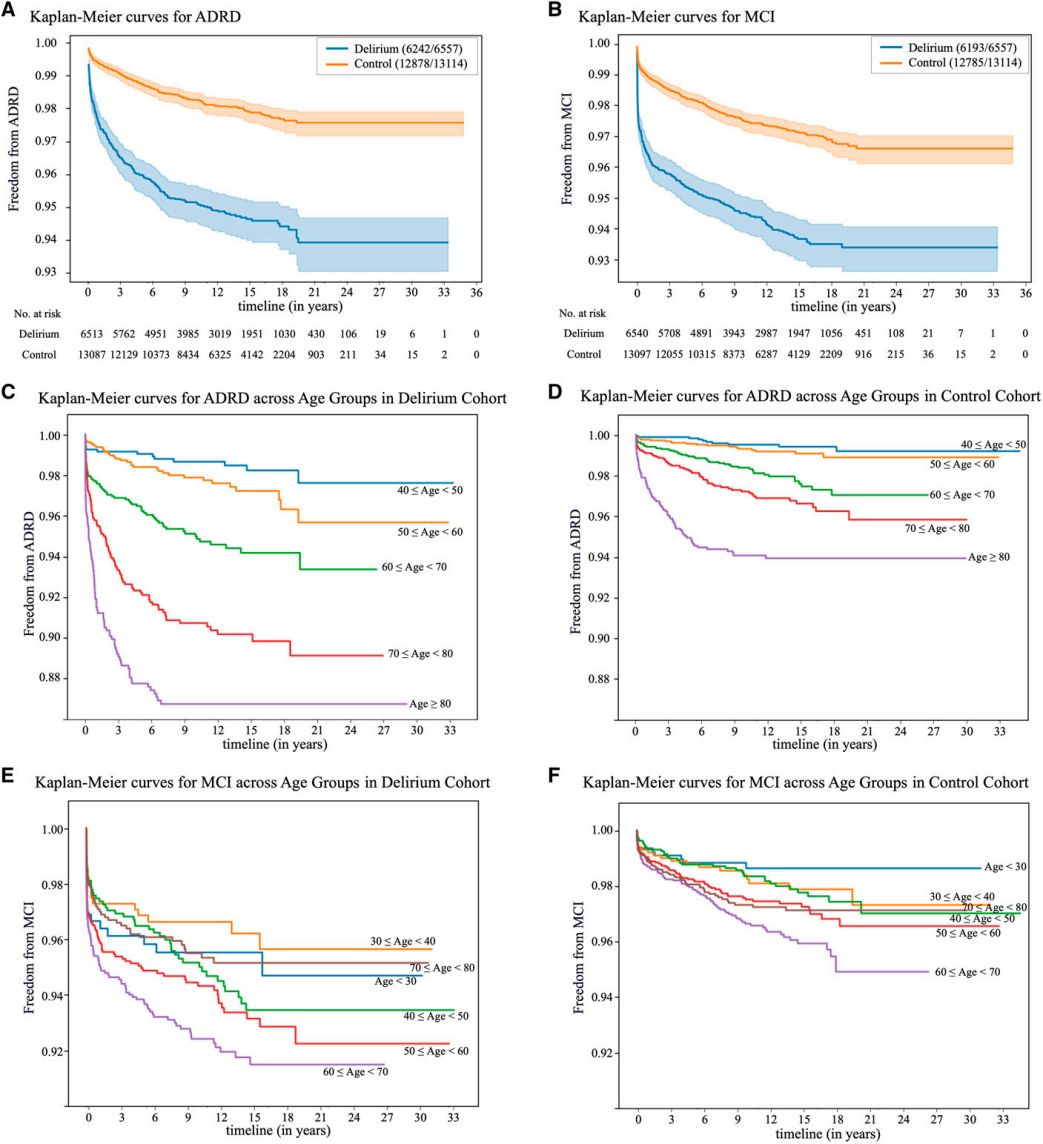
Survival analysis and age-stratified risk of mild cognitive impairment (MCI) and Alzheimer’s disease and related dementias (ADRD). (*A* and *B*) KM survival curves comparing the incidences of ADRD (*A*) and MCI (*B*) between the delirium and control groups over the follow-up period. The figure shows the probability of remaining free from ADRD or MCI. In the key, *n*_1_/*n*_2_ indicates the number of patients free from the risk (*n*_1_) relative to the total number of patients (*n*_2_) in each group. The numbers along the bottom axis represent the count of patients still under observation at every three-year follow-up interval for each group. (*C* and *D*) KM curves illustrating the age-stratified risk of ADRD in the delirium and control groups, respectively. (*E* and *F*) Age-stratified KM curves for MCI risk in the two groups. The *P* values for group differences in MCI and ADRD risk across all age categories (*A* for ADRD risk and *B* for MCI risk) and within each age category (*C* for delirium ADRD risk, *D* for control ADRD risk, *E* for delirium MCI risk, and *F* for control MCI risk) are all <0.001. KM = Kaplan-Meier.

An absolute standardized mean difference of 0.012, well below the 0.1 threshold, indicated well-balanced propensity score matching. The Kaplan-Meier analysis showed that the delirium group had significantly lower probabilities of freedom from ADRD and MCI compared with the control group (*P* < 0.001) (Figures 1A and 1B). The FG hazard ratios (HRs) were 2.20 (95% confidence interval [CI], 1.89–2.56) for MCI and 2.66 (95% CI, 2.24–3.16) for ADRD, indicating that the delirium group had higher odds of developing MCI and ADRD than the control group. In addition, the delirium group developed MCI 1.03 years earlier (RMST 31.06 vs. 32.09 yr) and ADRD 1.06 years earlier (RMST 31.29 vs. 32.35 yr) than the control group.

Delirium was associated with higher ADRD risk in all age groups ≥40 years (Figures 1C and 1D), with FG HRs as follows: 40–49 years, 3.08 (95% CI, 1.37–6.91; *P* = 0.006); 50–59 years, 3.36 (95% CI, 1.92–5.89; *P* < 0.001); 60–69 years, 2.83 (95% CI, 1.96–4.07; *P* < 0.001); 70–79 years, 3.33 (95% CI, 2.42–4.57; *P* < 0.001); and ≥80 years, 1.97 (95% CI, 1.46–2.65; *P* < 0.001). Delirium was also linked to higher MCI risk for ages 18–79 (Figures 1E and 1F), with HRs as follows: <30 years, 3.46 (95% CI, 1.58–7.54; *P* = 0.002); 30–39 years, 2.05 (95% CI, 1.08–3.91; *P* = 0.029); 40–49 years, 2.77 (95% CI, 1.81–4.22; *P* < 0.001); 50–59 years, 2.53 (95% CI, 1.83–3.51; *P* < 0.001); 60–69 years, 2.33 (95% CI, 1.75–3.09; *P* < 0.001); and 70–79 years, 1.71 (95% CI, 1.16–2.54; *P* = 0.007).

Delirium duration in the delirium group was not associated with higher ADRD risk (FG HR, 1.01 [95% CI, 0.99–1.03]; *P* = 0.31) but did increase MCI risk (FG HR, 1.02 [95% CI, 1.00–1.04]; *P* = 0.01). In subgroup analysis, patients discharged to short-term or long-term care had higher ADRD risk than those discharged home (FG HRs, 2.27 [95% CI, 1.38–3.72] and 1.92 [95% CI, 1.26–2.91], respectively; *P* < 0.001 for both), whereas MCI risk did not vary by discharge disposition. Even after including discharge disposition as a covariate, delirium remained significantly associated with increased risks of both ADRD (FG HR, 3.61 [95% CI, 2.49–5.24]) and MCI (FG HR, 2.96 [95% CI, 2.06–4.26]) (*P* < 0.001 for both).

## Discussion

Delirium increased the risk of MCI and ADRD, regardless of discharge disposition, and advanced MCI onset by 1.03 years and ADRD onset by 1.06 years. This suggests underlying neuropathological processes accelerating cognitive decline. Subgroup analyses showed consistently higher MCI and ADRD risks across age groups, except for no significant MCI change in patients ≥80 years and no significant ADRD difference in those <40 years.

Our retrospective design and reliance on EHR data may have missed prebaseline MCI/ADRD. EHR phenotyping cannot fully capture delirium subtypes or dose–response relationships (8), and changing diagnostic criteria over three decades may have affected our results. The OHDSI delirium phenotype lacks published performance metrics, which may reduce precision. Unmeasured factors such as family history, social and environmental influences, or detailed cognitive assessments could also shape our findings. Nonetheless, patients with delirium or those discharged to care facilities may benefit from more frequent follow-up for MCI or ADRD risk.
